# Characterization of Toxic Metals in Tobacco, Tobacco Smoke, and Cigarette Ash from Selected Imported and Local Brands in Pakistan

**DOI:** 10.1155/2014/413614

**Published:** 2014-02-02

**Authors:** Huma Ajab, Asim Yaqub, Salman Akbar Malik, Muhammad Junaid, Sadia Yasmeen, Mohd Azmuddin Abdullah

**Affiliations:** ^1^Department of Chemical Engineering, Universiti Teknologi PETRONAS, Bandar Seri Iskandar, 31750 Tronoh, Perak Darul Ridzuan, Malaysia; ^2^Department of Civil Engineering, Universiti Teknologi PETRONAS, Bandar Seri Iskandar, 31750 Tronoh, Perak Darul Ridzuan, Malaysia; ^3^Department of Biochemistry, Faculty of Biological Sciences, Quaid-i-Azam University, Islamabad 45320, Pakistan; ^4^Information Technology Department, University of Haripur, Pakistan

## Abstract

In this study, concentrations of Cd, Ni, Pb, and Cr were determined in tobacco, tobacco smoke-condensate, and cigarette ash for selected brands used in Pakistan. Smoking apparatus was designed for metal extraction from cigarette smoke. Samples were digested through microwave digester and then analyzed by flame atomic absorption spectrophotometer (FAAS). Higher concentration of Ni was detected in imported brands than the counterparts in the local brands. Pb levels were however higher in local brands while significant concentration of Cd was observed in both brands. For Cr, the level in tobacco of local brands was higher than their emitted smoke, whereas imported brands showed higher level in smoke than in tobacco. The cigarette ash retained 65 to 75% of the metal and about 25 to 30% went into the body. While this study revealed the serious requirement to standardize the manufacturing of tobacco products, more importantly is the urgent need for stronger enforcements to put in place to alert the general population about the hazardous effects of cigarettes and the health risks associated with these toxic metals.

## 1. **Introduction**

Cigarette smoking is a leading cause of various cancers and diseases associated with inhalation of toxic chemical substances produced by pyrosynthesis or liberated during combustion. Tobacco smoke is a source of toxic substances that constitute one of many classes of carcinogens, toxins, and addictive substances [1]. Various toxic heavy metals such as cadmium (Cd), lead (Pb), nickel (Ni), and chromium (Cr) from the smoke not only pose environmental hazard, but also may change from one form to another and persist in the environment. The tobacco smoke and ash could be significant contributor to metal load in the soil, air, and water systems in addition to the adverse human health effects via direct tobacco consumption [2].

Cd and Pb investigated in tobacco smoke have been classified as Group I and Group IIA carcinogens [3–5]. Pb toxicity has been reported to cause anemia, headache, irritability, and renal damage [6]. Cd is also poisonous even at lower doses in humans as it disrupts different biological systems like lungs, liver, and kidney [7–9]. In recent years, contamination by hexavalent Cr has become a major concern as it is a highly toxic carcinogen and its elevated level in humans may cause death [10]. Similarly, Ni has been found to be responsible for quite a number of ailments including dermal, lung, and nasal sinus cancers [11].

It is of paramount importance to evaluate the distribution of these metals in tobacco, its smoke, and ash to give indication to its presence and health hazard. Much work has been done on evaluation of metals in tobacco, but little attention has been made on the effects of smoke and ash especially on “passive” smokers. The present study correlates the metals concentration in tobacco, its smoke, and ash where the fraction residing in the mainstream smoke actually represents a smoker's primary exposure route.

The objectives of the current study were to evaluate the concentrations of Cd, Ni, Pb, and Cr in tobacco, tobacco smoke, and tobacco ash of local and imported cigarette brands and find significant differences and comparison between them. For the analysis of the present work, only popular cigarette brands were taken into consideration and standard analytical conditions were employed during the measurements.

## 2. Materials and Methods

### 2.1. Chemical and Reagents

All chemicals and reagents used were of analytical grade and purchased from E-Merck (Darmstadt, Germany). Nitric acid (10%) was used to soak glassware for 24 h followed by rinsing with deionized water and then oven drying [12]. For digestion purpose, 65% HNO_3_ and 60% HClO_4_ were used. Stock solutions containing 1000 ppm of metals were used as standards.

### 2.2. Microwave Digestion

**Figure 1:**
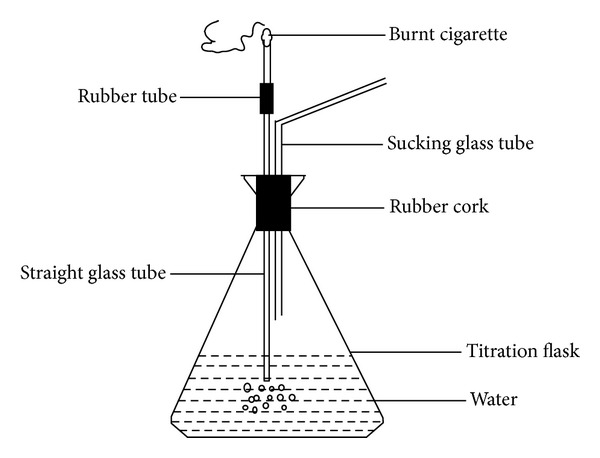
Smoking apparatus.

Microwave digestion technique was used for digestion of samples, so that accuracy could be attained, with minimum chances of contamination with complete digestion of analytes [12, 13]. An other key point was the arrangement of small smoking apparatus (as shown in Figure 1) that was inhaled by the volunteers. The dissolved metals in the form of smoke condensate from mainstream smoke of cigarettes were measured.

### 2.3. Preparation of Tobacco Samples

The method for preparation of tobacco samples was as reported before [12]. As shown in Table 1, a total of twenty cigarette brands (10 local and 10 imported) commonly used in Pakistan were purchased from the local market. The average weight of each cigarette brand was determined by weighing 5 sticks of each brand before and after removing the filters using Sartorius Analytical Balance (ISO 9001).

### 2.4. Preparation of Smoke Samples

An aliquot of 50 mL of double distilled, deionized water was added into the acid washed titration flask. A rubber cork with two holes was fitted into the mouth of titration flask. In one hole, a bent glass tube (sucking tube) was inserted such that its lower end is above the water level and in the other hole, a straight glass tube was inserted whose lower end was immersed into the water. At the top end, small rubber tube able to hold a burning cigarette was attached (Figure 1).

The volunteers were asked to smoke 5 sticks of each brand from the same packet of cigarettes wherefrom the tobacco samples were used. Volunteers sucked from the bent glass tube, so that the mainstream smoke (smoke inhaled by the smoker) of cigarette from the straight glass tube is first drawn into the water and then passed to the mouth of the volunteer through the bent tube. In this way the metals in the form of smoke condensate representing metals otherwise inhaled by the smoker were collected. The smoke condensate and dropped ash of the same cigarette were collected and analyzed. The volunteers were allowed to smoke freely as per their own will without changing their daily routine.

An aliquot of 20 mL of fresh smoked condensate was digested with 5 mL HNO_3_ with the program of microwave digester as shown in Table 2.

### 2.5. Preparation of Ash Samples

All steps were kept alike for tobacco sample preparation but the dropped ash was digested based on the program as shown in Table 3.

### 2.6. Metals Concentration

For the accuracy of the analytical results by AA 240 FS fast sequential atomic absorption spectrometer (Varian Australia Ltd.), at least one reagent blank was analyzed. A verification of the samples preparation and measurements was also carried out by analysis of lyophilized blood samples (batch 1701–1703) provided by Jasper Kristiansen (AMI, Denmark).

## 3. Results and Discussion

Table 4 shows the average moisture content percentage (MC%) in local and imported cigarette brands. The average moisture content of local cigarette brands in tobacco was 10.3% and ranges from 4.9 to 17.2% (*μ*g/cigarette). The maximum value was found in Gold Flake (17.15%) and the minimum in Red & white (4.902%). The average moisture content of imported cigarette brands in tobacco was 8.9% and ranges from 4.5 to 13.1% (*μ*g/cigarette). The maximum value was found in Rothmans (13.8%) and the minimum in Mild Seven (4.483%). MC% in tobacco is an important quality characteristic.

**Figure 2:**
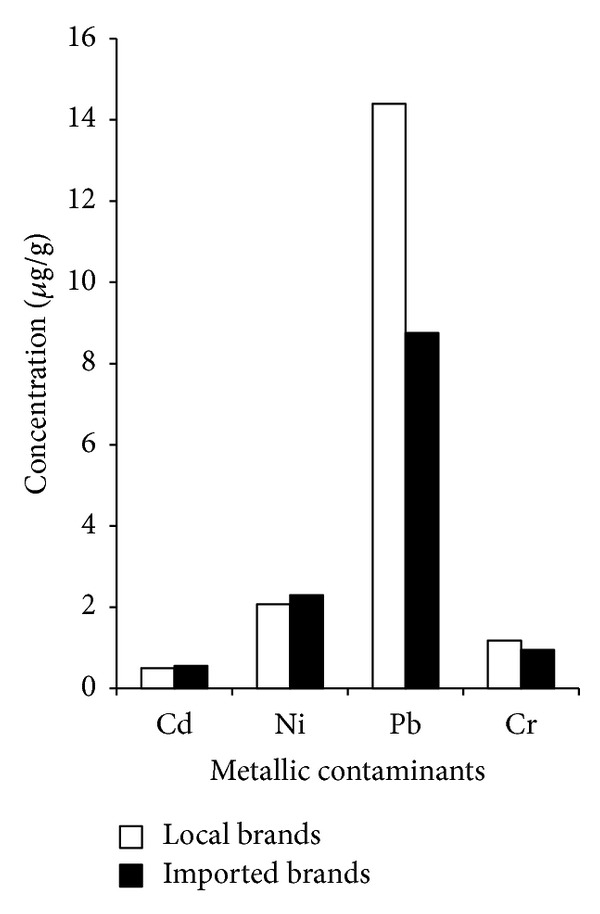
Comparison between local and imported cigarette brands in tobacco.

**Figure 3:**
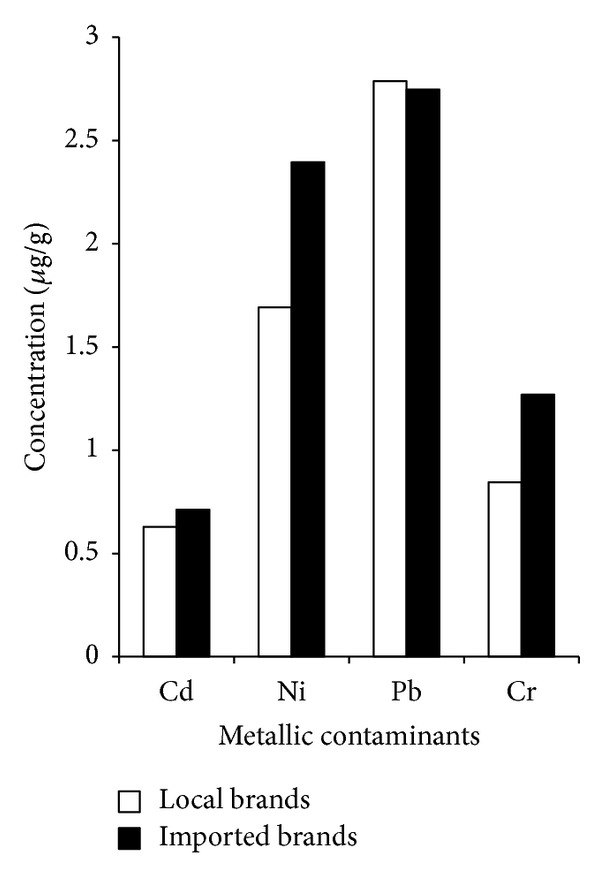
Comparison between local and imported cigarette brands in smoke.

**Figure 4:**
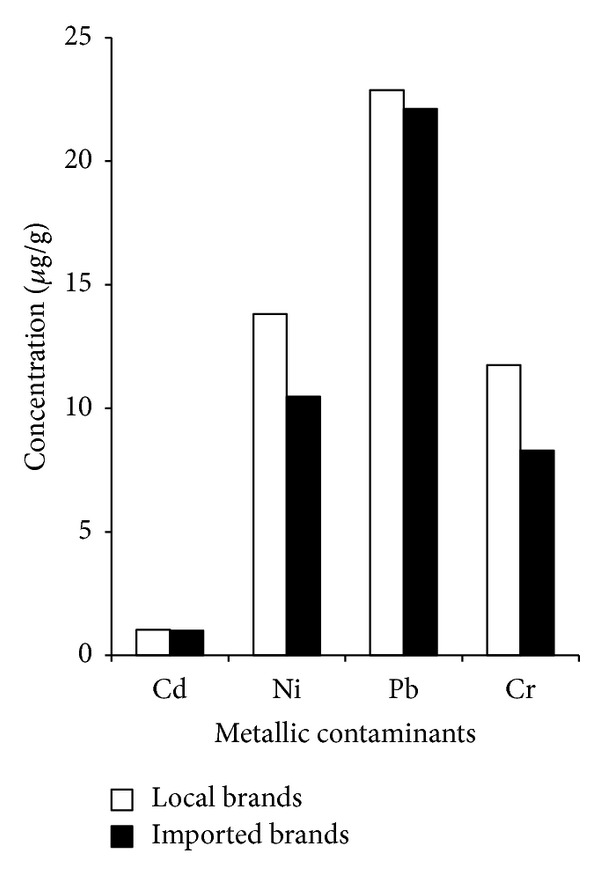
Comparison between local and imported cigarette brands in ash.

Comparisons between metal contaminants in local and imported cigarette brands in tobacco, smoke, and ash are given in Figures 2, 3, and 4, respectively. The results for Cd concentration demonstrate a descending order of the following: ash > smoke > tobacco both for local as well as for imported cigarette brands. In case of Ni, local and imported cigarette brands exhibited the following descending orders: ash > tobacco > smoke and ash > smoke > tobacco, respectively. The values of local and imported brands for the Pb concentrations showed the following trend: ash > tobacco > smoke. The concentration of Cr in local and imported cigarette brands exhibited the following orders: ash > tobacco > smoke and ash > smoke > tobacco, respectively. Concentrations of Cd and Pb in tobacco were comparable with previous study [12].

The metal-to-metal correlation statistics as in Tables 4 and 5 (correlation was considered significant at 0.05 level) for tobacco in imported cigarette brands indicated strong positive correlations for Ni–Cd (*r* = 0.632) and Pb–Cd (*r* = 0.543). In case of smoke from local brands, strong positive correlation for Cr–Ni (*r* = 0.687) was found while the smoke from imported brands showed strong positive correlations for Pb–Cd (*r* = 0.704) and Cr–Ni (*r* = 0.644). Pb metal was strongly and positively correlated with Cd (*r* = 0.542) in the ash of local brands and ash of imported brands that revealed strong positive correlation for Cr–Ni (*r* = 0.653). All other cases exhibited positive as well as negative weak correlations. Similar observation on significant correlation between Ni and Cr has been reported [14].

Elevated Pb levels in tobacco of local brands were detected. According to WHO estimation, smoker inhales 2–6% of Pb (WHO 1989) [15]. Present findings both in local (19%) and imported (28%) cigarette brands showed significant deviation from WHO estimation. The Cd levels are in good agreement with Watanabe et al. [16] which report the Cd content in cigarettes sampled from various countries ranging from 0.290 to 3.338 *μ*g/g. Higher concentration of Cd was observed in tobacco of imported brands while higher Ni was observed both in tobacco and smoke in imported brands. Present results were in good agreement with previous report [17] that considerable concentrations of Ni in burning cigarette are transferred to mainstream smoke.

The level of Cr in tobacco of local brands was higher but with correspondingly fewer fractions that appeared in smoke. Imported brands had greater concentration of Cr in smoke due to the release of Cr from burning of cigarette wrapping paper and filter because they too contribute metals to mainstream smoke. The distribution of Cd in smokes of both local and imported brands was higher than the respective tobacco. It is likely that the transport of particulate mass through the tobacco rod and filter is influenced by the cigarette's design feature. It can be concluded that levels of heavy metals in mainstream smoke correlate well with filter ventilation designs [18]. The ash showed that 65–75% of the mass of the metals was retained in cigarette ash. Ashes are therefore also the possible source of contaminants requiring proper disposal in the environment.

In cigarette manufacturing, as much as 600–1400 additives are used, many of which contain trace metals [19]. Besides additives, the main sources of metallic contaminants are the paper and the filter. Certain levels of metals may be higher or lower in tobacco grown in a given location, depending on geographical location, industrial or mining activities, and agronomic practices [18]. The excessive use of fertilizers, pesticides, and irrigation with residual water is among the common causes of contamination of raw foodstuff and tobacco leaves. Tobacco plant grown in soils having higher lead levels has been reported with corresponding higher lead levels in the smoke particulate [17]. The high concentrations of Cd in tobacco leaves may result from the widespread use of chemical fertilizers [20, 21]. The tobacco plant absorbs Ni and other toxic metals mostly from soil, pesticides, and other fertilizing products.

Current interest in the physiological effects of smoking makes it desirable to study metallic contaminants not only in tobacco but also in smoke condensate and ash. It is critical to have selective, accurate, and sensitive methods for measuring toxic metals in tobacco and smoke. The present study explored a new and scientifically sound basis to evaluate heavy metal contaminants in cigarettes as there was no sufficient data on metallic contaminants in tobacco, specifically in smoke condensate and ash of cigarette brands used in Pakistan. It is confirmed that cigarette smoking is a source of many toxic metals and their quantitative distribution is mostly well above the safer limit as established by WHO. The elevation of metal contamination levels in cigarette brands available in Pakistan is possibly due to the growing conditions of tobacco crop, and industrial (processing) and mining activities. The comparative evaluation puts concentration of Pb metal in tobacco, smoke, and ash of local brands at the top of the rank. The monitoring of heavy metals during growing, processing, and smoking of tobacco is therefore essential for protection of the environment and health. This finding should alert the health authorities to formulate necessary policy to keep the environmental load within tolerable limits. With health risks associated with these toxic metals, there should be a stricter quality control over the monitoring of heavy metals during growing, processing, and smoking of tobacco to minimize health hazards to both active cigarette smokers and to those exposed to the tobacco smoke.

## Figures and Tables

**Table 1:** Moisture contents of local and imported cigarette brands.

Local brands	Moisture content (%)	Imported brands	Moisture content (%)
Gold Leaf	10.62	Marlboro	9.636
Diplomat	10.57	Rothmans (King size)	13.08
K 2 (King size)	10.94	Benson & Hedges	10.56
Embassy (Kings)	9.985	Mild Seven	4.483
Red & white (King size)	4.902	Pine (Menthol lights)	5.632
Morven Gold	5.800	More (International)	11.94
Royals (Filter)	15.48	Business club	10.32
Gold Flake	17.15	Dunhill (International)	5.085
Park Lane (Special)	5.181	Pine Lights	9.804
Capstan (International)	12.20	Fisher	7.931

Average	10.28%	Average	8.847%

**Table 2:** Digestion program for smoke samples.

Power (watt)	Max. %	*t* (mins)	*T*°C	Hold (mins)
600	50	10	150	10
600	50	10	100	10

**Table 3:** Digestion program for ash samples.

Power (watt)	Max. %	*t* (mins)	*T*°C	Hold (mins)
1200	100	20	200	10
600	50	10	170	10

**Table 4:** Analysis of variance (ANOVA) for local cigarette brands in tobacco, smoke, and ash.

	*n* = 30, mean ± SD (µg/g)			*P* value	Remarks
	Tobacco	Smoke	Ash		
Cd	0.501 ± 0.120	0.629 ± 0.132	1.029 ± 0.054	0.000**	Significant
Ni	2.076 ± 1.113	1.691 ± 0.451	13.81 ± 1.591	0.000**	Significant
Pb	14.39 ± 4.986	2.787 ± 0.835	22.88 ± 20.03	0.03*	Significant
Cr	1.178 ± 0.196	0.844 ± 0.521	11.75 ± 1.365	0.000**	Significant

*Significant difference (*P* < 0.05).

**Highly significant difference (*P* < 0.01).

**Table 5:** Analysis of variance (ANOVA) for imported cigarette brands in tobacco, smoke, and ash.

	*n* = 30, mean ± SD (µg/g)			*P* value	Remarks
	Tobacco	Smoke	Ash		
Cd	0.559 ± 0.141	0.712 ± 0.112	0.991 ± 0.105	0.000**	Significant
Ni	2.294 ± 1.956	2.394 ± 1.728	10.47 ± 3.200	0.000**	Significant
Pb	8.749 ± 3.111	2.800 ± 0.761	22.11 ± 16.45	0.000**	Significant
Cr	0.951 ± 0.267	1.269 ± 0.737	8.294 ± 2.484	0.000**	Significant

*Significant difference (*P* < 0.05).

**Highly significant difference (*P* < 0.01).
